# Recognition and Avoidance of Contaminated Flowers by Foraging Bumblebees (*Bombus terrestris*)

**DOI:** 10.1371/journal.pone.0026328

**Published:** 2011-10-24

**Authors:** Bertrand Fouks, H. Michael G. Lattorff

**Affiliations:** Institute of Biology, Molecular Ecology, Martin-Luther University Halle-Wittenberg, Halle, Germany; AgroParisTech, France

## Abstract

Bumblebee colonies are founded by a single-mated queen. Due to this life history trait, bumblebees are more susceptible to parasites and diseases than polyandrous and/or polygynous social insects. A greater resistance towards parasites is shown when the genetic variability within a colony is increased. The parasite resistance may be divided into different levels regarding the step of the parasite infection (e.g. parasite uptake, parasite intake, parasite's establishment in the nest, parasite transmission).We investigate the prophylactic behaviour of bumblebees. Bumblebees were observed during their foraging flights on two artificial flowers; one of these was contaminated by *Crithidia bombi*, a naturally occurring gut parasite of bumblebees (in a control experiment the non-specific pathogen *Escherichia coli* was used). For *C. bombi*, bumblebees were preferentially observed feeding on the non-contaminated flower. Whereas for *E. coli*, the number of visits between flowers was the same, bumblebees spent more time feeding on the non-contaminated flower.These results demonstrate the ability of bumblebees to recognise the contamination of food sources. In addition, bumblebees have a stronger preference for the non-contaminated flower when *C. bombi* is present in the other flower than with *E. coli* which might be explained as an adaptive behaviour of bumblebees towards this specific gut parasite. It seems that the more specific the parasite is, the more it reduces the reward of the flower.

## Introduction

Among all metazoans, parasites and diseases represent a strong threat reducing the life time and the fitness of an organism [1], and also a strong evolutionary force [2]. When a parasite is specific to a host, the relation, regarding the evolution, between these two species is linked and may lead to co-evolution. This co-evolution between a parasite and a host results in an arms race [3], [4]. The host will tend to evolve to reduce the effects of the parasites on themselves. Many levels are involved in resistance to a parasite [5]. The first one is the reduction of parasite uptake, allowing individuals to avoid the parasite. The second one is the non-intake of the parasite resulting to a protection against the intrusion of the parasite in the organism. The third one is the reduction of parasite loads inside the host and even the complete elimination of the parasite. The last level is the prevention of transmission of the parasite in order to avoid secondary infection and the infection of the conspecifics.

Eusocial insects provide a rich and stable environment for parasites [6]. Indeed, living in a closed nest with a large amount of nest-mates provides a parasite with a lot of individuals to infect in a close and tiny spatial environment. The homeostatic nest conditions may additionally improve parasite survival.

One explanation to the evolution of polyandry in social insects is to reduce the parasite load [6]. Indeed, several empirical studies have proved that increasing genetic diversity among nest-mates diminishes the parasite load within the colony [7], [8], [9], [10], [11], [12], [13]. Two factors are claimed to be responsible to this. First, the spread of a parasite within a colony is reduced when the worker genotype variability is high; due to the host-parasite genotype-genotype interactions [14]. Secondly, the increase of genetic variability within a colony results in an increased likelihood for the presence of individuals resistant to parasites; since different genotypes vary in their resistance to parasites [13], [15]. Monandrous and monogynous species seem so to be more susceptible and defenceless to parasites, when they are established in the nest [10].

Parasites in social insects appear to be a great concern in ecology since they are responsible for the world wide decline of pollinators; especially in bees [16], [17].

In bumblebees, the colony is founded by one single-mated queen [18], [19]. This reduces the genetic variation within a colony thereby increasing the risk of the spread of a parasite within the colony. Regarding this, when the parasite is established in one individual, it can spread easily within the colony and affect the entire colony. The most adaptive strategy to resist parasite in bumblebees should be the avoidance of parasite uptake or intake into the colony.

One of the most widespread parasites in bumblebees is *Crithidia bombi,* a trypanosome gut parasite. *C. bombi* may cause a decrease of colony efficiency, a higher mortality of workers and/or a delay on the production of the reproductive caste [20]. Transmission of *C. bombi* might occur vertically, but also horizontally by foragers on flowers [21]. *C. bombi* may be transmitted to other conspecific, even allospecific pollinators, via shared used of flowers [21]. The presence of this parasite on flowers has been recorded [21]. The ingestion of this parasite results in a rapid immune response. The immune genes are up regulated 24 hours post infection [22]. The same pattern has been shown to occur with non specific parasites (*E. coli*) [23]. This immune response is known to reduce the learning ability of free flying bumblebees [24]. *C. bombi* is further known to change the foraging behaviour of bumblebees. When they are infected with *C. bombi*, they spend more time foraging due to a reduce ability to handle the flower [25]. Bees infected with *C. bombi* reject more flowers and fall more often from the flower [25].

To test, whether bumblebees are adapted to resist against a specific parasite and if avoidance behaviour was selected against contaminated flowers; bumblebee colonies were observed during a foraging test. Bumblebees were marked individually and were given a choice between two flowers: one where the pathogen is present in the nectar referred later as “contaminated” and the other where the pathogen is absent from the nectar referred as “non-contaminated”. This experiment was repeated with different pathogens: a common, non-specific pathogen *Escherichia coli* and the specific parasite *Crithidia bombi*. The number of visits, the visit duration and the individual feeding on each flower were recorded and compared.

## Materials and Methods

### *Bombus terrestris*

To test the ability of bumblebees to recognise contaminated flowers, the foraging of bumblebee workers from a commercial colony was observed on artificial flowers under semi-natural conditions within a tent (4 m×5 m×2 m) placed outdoors. Four replicates were made for *E.coli* and *C. bombi* experiments with separate colonies. The bumblebee colony was placed on a chair at a distance of two meters from the flowers. The bumblebees were kept in their original colonies and were provided only with pollen *ad libitum*, foraging was for sugar or honey water. The flowers were equidistant from the colony and were placed at 10 cm apart from each other. The artificial flowers were built from a model of the umbel flower from Jordan & Harder 2006[26] and consisted of twelve Eppendorf® tubes (0.6 ml) wrapped in blue paper and pinned on a cardboard disc (Ø12 cm) by an insect pin. Before the recording, bumblebees were trained to forage on the flowers. During training, the flowers were filled with a mixture of honey and 50% sucrose solution (v/v). The training occurred over 3 to 5 days depending on the frequency of individuals foraging. After training, the observations were started with one of the flowers contaminated by a pathogen. During the experimental period, the flowers were filled with the same mixture as during the training, when no observation was taking place. Bumblebee workers were marked individually using Opalithplättchen (I.D.) glued (ApisPro®) to their thoraces. The individual I.D., the number of visits and the visit duration were recorded for each flower. When individuals lost their marking, they were recorded as unknown individuals and were attributed a different number for each visit. The recording time started when the bumblebee began feeding on the flower and stopped when they departed. When the identification of individual's marking was impossible (staying on the flower less than 2 s), the visit was discarded.

### *Escherichia coli*

The first experiment was conducted by infecting one flower with *Escherichia coli*, a non-adapted pathogen. *E. coli* (strain JM109 from Promega®) was cultivated in 30 ml LB medium as over night culture at 37°C. After counting with a Fuchs-Rosenthal counting chamber (Roth, Karlsruhe, Germany) according to standard protocols, the cell culture was centrifuged 20 min at 2000 rpm. The LB medium was extracted and the pellet was mixed with a 50% sucrose solution (v/v) in order to get a concentration of *E. coli* at 10^5^ cells*ml^−1^. Four commercial bumblebee colonies (Koppert Biological System®) were used containing each 70 to 150 workers. The recording occurred 4 hours per day over a period of 4 days. The flowers were switch every hour.

### *Crithidia bombi*

In a second experiment, *Crithidia bombi* was used to infect one of the flowers. *C. bombi* were extracted from wild bumblebees' guts from Halle (Germany) (No specific permits were required for the extraction of *C. bombi* from wild bumblebees. The sample was on an open area not privately owned and not protected in any way, and concerns only bumblebee workers which are not considered as an endangered or protected animal.). One strain of *C. bombi* cells was cultivated and counted according to the methods developed by Popp & Lattorff 2010[27]. The cell culture of *C. bombi* was centrifuged for 20 min at 2000 rpm. The pure medium was discarded and the pellet was diluted in 50% sucrose solution (v/v) in order to get a concentration of 10^4^ cells*ml^−1^. Four commercial colonies were used (2 from Koppert Biological System® and 2 from Biobest Biological System®) containing each 70 to 150 workers. We used the two commercial sources to test for differences between maintained populations (one population from Central Europe and one from South Europe; possibly different subspecies). The visits were recorded until the total number of visits was 350 for each colony; the flower position was switched 4 times per day in order to account for any side preference of the foraging workers and to get the same number of visits for each flower position per day. For three colonies, the time of recording was 3 days and for the last colony the record was running for a total of 6 days.

### Control

A control experiment was made to certify the absence of influence of the culture medium on the bumblebee foraging decisions. One commercial colony (Koppert Biological System®) was used for the record and one flower received a mixture of medium and sugar water (concentration: 1.34% according to twice the concentration of medium expected in the contaminated sucrose solution of both other experiments). Behavioural recordings were done according to the methods described for the *C. bombi* experiment.

### Statistical analyses

The avoidance behaviour exhibited by bumblebees was expected to be specific and so should be more frequent when a specific pathogen of bumblebees was present in a flower. Hence the proportion of visits on the uncontaminated flower was compared between the different pathogens. We assigned the value 1 for a visit on the uncontaminated flower and 0 for a visit on the contaminated flower. The proportion of visits on the uncontaminated flower was analysed between the different experiments by a generalized linear mixed effect model with a binomial distribution including as a fixed factor the pathogen type (*E. coli*, *C. bombi*, and control) and individual and colony I.D., and day of recording as random factors to account for pseudo-replication between days and, between and within colonies.

#### *E. coli*

The data for feeding duration for each set up were log transformed and analysed with a generalized linear mixed effect model [28], [29] including the individual and colony I.D., and the day of recording as a random factors to account for pseudo-replication between days and, between and within colonies. The contamination of the flower (contaminated or not) and the position (left or right) were included as fixed factors in all models. The distribution of all response variables and their residuals were inspected for symmetry. Factor levels were reduced from the full model by stepwise deletion (model simplification following Crawley 2005 [30]).

The number of visits was analysed by a generalized linear mixed effect model with a Poisson distribution including as explanatory factors: the contamination, the position; and as random factor: the individual and colony I.D., and the day of recording to account for pseudo-replication between days and, between and within colonies. Factor levels were reduced from the full model by stepwise deletion (model simplification following Crawley 2005 [30]). Furthermore when a model was better than the null model, another generalized linear mixed effect model was built. In order to test how the proportion of uncontaminated flower visitation changes over days and in regard to the position of the flower, the proportion of visits on the uncontaminated flower was analysed using a generalized linear mixed effect model with a binomial distribution. The day of recording and the position of the flower were included as fixed factors while the individual and colony I.D., and day of recording as a random factors to account for pseudo-replication between days and, between and within colonies. Factor levels were reduced from the full model by backward stepwise deletion (model simplification following Crawley 2005 [30]).

#### *C. bombi*

The same statistical method applied for *E. coli* was used for the visit duration and the preference toward a flower in the *C. bombi* experiment. When testing for the distributions of uncontaminated flower visitation over days and position, a third fixed factor was added to the model: origin of the colony (i.e., company).

In addition, to understand the decision making at an individual level in the *C. bombi* experiment, individuals with different total number of flights (Fig. S1) were classified in different groups: individuals with less than or equal to 5 flights the naive bees [31], [32] and individuals with more than or equal to 10 flights the experienced bees. Individuals recorded as unknown were excluded from this analysis.

The naive bees were used to analyse if the individuals were able to recognise and avoid the contaminated flower without experience. So the number of visits between the contaminated and uncontaminated flowers was compared using a Mann-Whitney-U-test.

The experienced bees were further divided in two groups: the rare (10 to 24 flights in total) and the frequent flyers (>25 flights in total). The proportion of visits on the non-contaminated flower was compared between these two groups on each day with a Mann-Whitney U test. In addition, the proportion of visits on the non-contaminated flower for each group was compared between days using a Friedman ANOVA and Kendall coefficient of concordance test.

##### **Control**

The same statistical method applied for *E. coli* was used for the control experiment without colony as random factor.

## Results

**Figure 1:**
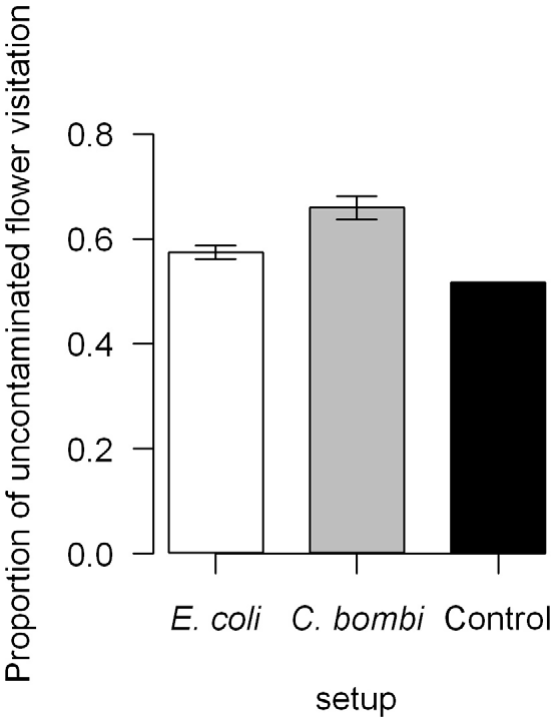
Proportion of non-contaminated flower visitation between experiments. The bars represent the means between the different colonies and their 95% confidence interval. The foragers were feeding more often on the non-contaminated flower when the other one was contaminated by a pathogen. This proportion increased when the other flower was contaminated with *C. bombi* (GLMM: *p*<0.001; *C. bombi* vs control: *p*<0.001, *C. bombi* vs *E. coli*: *p*<0.001, *E. coli* vs control: *p*<0.01).

The proportion of visits on the uninfected flower is higher for the *C. bombi* experiment than for the *E. coli* one. For the control experiment, this proportion was lower than for either of the other experiments (Fig. 1, GLMM: *p*<0.001). This highlights an increased preference, or a better ability to avoid the contaminated flower, in the presence of *C. bombi* than *E. coli* (*C. bombi* vs control: *p*<0.001, *C. bombi* vs *E. coli*: *p*<0.001, *E. coli* vs control: *p*<0.01).

### *Escherichia coli*

**Figure 2:**
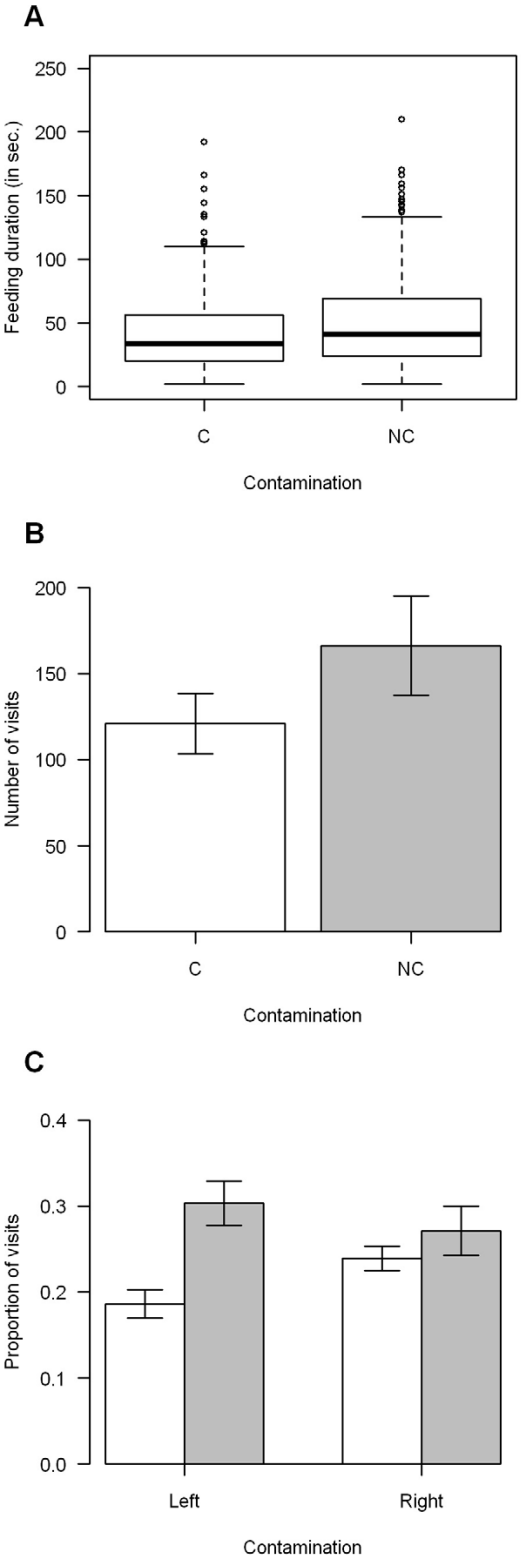
Feeding duration, flower preference and proportion of uncontaminated flower visitation for *E. coli* experiment. A) Feeding duration on both flowers with and without the presence of *Escherichia coli* (n = 1150), B) Visit duration on both flowers with and without the presence of *Escherichia coli* (n = 1150), C) Proportion of non-contaminated flower visitation for *E. coli* experiment. C (in white) represents the presence of the parasite in the flower and NC (in grey) its absence. For the feeding duration, box plots depict median, interquatile range and non-outlier range; the dots represent the outliers. The bars represent the means between the different colonies and their 95% confidence interval. Foragers feed longer on the uncontaminated flower (GLMM: *p*<0.05), visit it more often (GLMM: *p*<0.01) and are more accurate when the flower is on left position (GLMM: *p*<0.001).

Bumblebees spent more time feeding on the non-contaminated flowers (Fig. 2a). For the visit duration the best model includes only the contamination as explanatory factor (GLMM: *p*<0.05). They also exhibited a preference for the non-contaminated flower. The number of visits observed was higher on the non-contaminated flower than on the contaminated one (best model includes only the contamination as explanatory factor GLMM: *p*<0.01, Fig. 2b). The bumblebees visited the non-contaminated flower more often when it was on the left position (best model includes only the position as explanatory factor, GLMM:, *p*<0.001; Fig. 2c).

### *Crithidia bombi*

**Figure 3:**
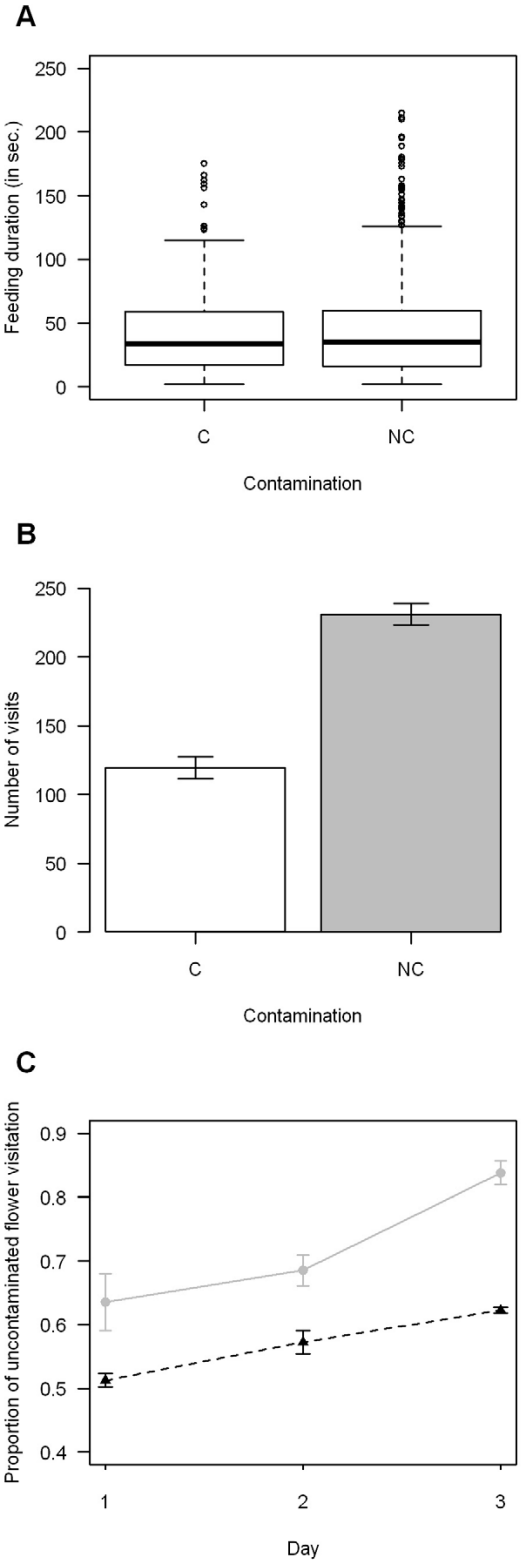
Feeding duration, flower preference and proportion of uncontaminated flower visitation for *C. bombi* experiment. A) Feeding duration on both flowers with and without the presence of *Escherichia coli* (n = 1400), B) Visit duration on both flowers with and without the presence of *Escherichia coli* (n = 1400), C) Proportion of non-contaminated flower visitation over days and between sympatric population (grey dot & continuous line) and allopatric population (black triangle & dashed line) for *C. bombi* experiment. C (in white) represents the presence of the parasite in the flower and NC (in grey) its absence. For the feeding duration, box plots depict median, interquatile range and non-outlier range; the dots represent the outliers. The bars represent the means between the different colonies and their 95% confidence interval. Foragers spend the same time feeding on both flowers (GLMM: *p* = 0.24), visit preferentially the uncontaminated flower (GLMM: *p*<0.001). The proportion of uncontaminated flower visitation increase over days and for the sympatric population this increase is stronger than for the allopatric population (GLMM: *p*<0.01; factor day: *p*<0.05, interaction between day and population's origin: *p*<0.01).

For the *C. bombi* contamination, bumblebees spent a similar amount of time foraging on the contaminated as on the non-contaminated one (GLMM: *p* = 0.24, Fig. 3a), but visit more frequently the non-contaminated flower (best model includes only the contamination as explanatory factor, GLMM: *p*<0.001; Fig. 3b). Moreover the number of visits increases over time and there is a different pattern of visitation between populations. Bumblebees exhibited a stronger preference for the non-contaminated flower. Indeed the best model includes the contamination as an explanatory factor. They also increased the number of visits on the non-contaminated flower over time (factor day: *p*<0.05), for the sympatric population this increase was stronger (interaction between day and population's origin: *p*<0.01). The best model included the day and the interaction between day and the population of origin as explanatory factors (GLMM: *p*<0.01; Fig 3c).

The naive bees are able to avoid the contaminated flowers since they visited more often the non-contaminated flower (M-W-U-test: Z = 5.74, *p*<0.001).

**Figure 4:**
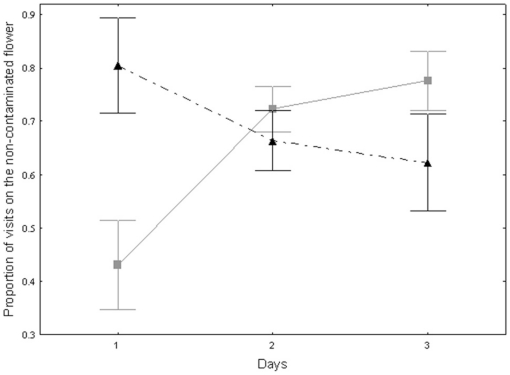
Proportion of visits on the flower without *Crithidia bombi* over days between the two groups of experienced foragers: frequent (n = 10) and rare flyers (n = 26). The black triangles and dash line represent the frequent flyers group and the grey squares and continuous line the rare flyers group. The symbol represent the mean and the bars the standard error. On the first day, the frequent flyers visited more often the flower without parasite than the rare flyers (M-W-U-test: Z = −2.40, *p*<0.05) but over days the rare flyers increased their proportion of visits on the flower where the parasite is absent to reach the same level than the frequent flyers (Friedman ANOVA: χ^2^ = 9.15, *p*<0.01; 2^nd^ day: M-W-U-test: Z = 0.77, *p* = 0.45; 3^rd^ day: M-W-U-test: Z = 1.49, *p* = 0.15).

Among the experienced bees, the frequent flyers have a better cognitive ability or sensory to recognise the contaminated flower than the rare flyers on the first day since they visited the non-contaminated food source more often (M-W-U-test: Z = −2.40, *p*<0.05, Fig. 4). Although after the first day, the rare flyers increase their number of visits on the non-contaminated flower (Friedman ANOVA: χ^2^ = 9.15, *p*<0.01, Fig. 4) and reach the same proportion of visitation on the non-contaminated flower as the frequent flyers (2^nd^ day: M-W-U-test: Z = 0.77, *p* = 0.45; 3^rd^ day: M-W-U-test: Z = 1.49, *p* = 0.15, Fig. 4). The frequent flyers showed no increase or decrease over time (Friedman ANOVA: χ^2^ = 4.26, *p* = 0.12, Fig. 4).

### Control

The medium has no influence on the feeding duration, or the number of visits, since the null model (without any explanatory factors) was not improved by adding explanatory factors (feeding duration: GLMM: *p* = 0.71; number of feeding events: GLMM, *p* = 0.33).

## Discussion

Our study assessed the ability of bumblebees to recognise food sources contaminated by an adapted parasite and a non-adapted microorganism under semi-natural conditions. The results highlight the existence of the avoidance behaviour during the foraging of bumblebees, a primitive eusocial insect. In addition, our results show that bumblebee foragers behave differently toward non-contaminated food sources and contaminated ones, with also a difference towards the type of contamination.

The *B. terrestris* population originating from the same region of Europe than the *C. bombi* lineage used for the experiment shows a better ability to avoid contaminated flower than the population allopatric with the parasite lineage. This seems to indicate an adaptation not only toward a specific parasite but also to a specific lineage of the parasite; maybe due to the host-parasite genotype-genotype interaction. This is seen at the immune response level where bumblebees show a greater resistance to specific strains of *C. bombi* [33]. An alternative explanation is a better ability of one population to avoid the contaminated flower compared to the other. It was argued and shown that avoidance behaviour in birds should be specific to a parasite species, but not a parasite strain [33], [34].

Bumblebees spent more time feeding on non-contaminated artificial flowers than on those contaminated by *E. coli* and visit the uncontaminated flower more often (Fig. 1a,b). Many theories on optimal foraging were tested in bumblebees and other pollinators, especially the marginal value theorem developed by Charnov in 1976 [35], [36], [37], [38], [39]. The results provided by these different experiments show that bumblebee foraging and patch departure follows a sub-optimal strategy [35], [36], [37], [38], [39]. To summarise briefly the strategy exhibited by bumblebees is to stay longer in large patches or patches providing a high reward. Patch departure happens with the decreasing reward of one flower or from the entire patch. In our experiment, we can consider one flower composed by 12 inflorescences as a patch. The flowers were filled appreciatively at a similar level and access to the “nectar” was similar between the two flowers. On one hand, this difference in feeding duration between the two flowers could be explained as a preference for the non-contaminated “nectar”, or as most rewarding "nectar". On the other hand, this difference in feeding duration could also result from the direct presence of the cells or the medium in the sugar water decreasing its energetic value for the bee. This last explanation seems to be contradicted by the *Crithidia* and control experiment where the presence of the gut parasite and the medium had no effect in the visit duration (Fig. 3a). The effect of position on the proportion of uncontaminated flower visitation could be due to a lateralization of the brain and behaviour in bumblebees [40].

The bees, having the choice between a contaminated food source by a specific gut parasite and a non-contaminated one, visit more often the non-contaminated flower (Fig. 3b). This reveals the clear ability of bumblebees to recognise and avoid sugar water contaminated by *Crithidia*. In a same context as above (comparing this foraging behaviour with the optimal foraging theorem) this result can be interpreted as flower constancy. Indeed, it was shown that a bee will prefer to visit a flower that she learnt to be rewarding than to spend time visiting other flowers [35], [36], [37], [38], [39], [41], [42]. The presence of *C. bombi* leads to a perceptive decrease of the reward provided by the sugar water to the bumblebees.

The comparison between the results of the experiments with *C. bombi* and with *E. coli* shows a degree of adaptation of bumblebees toward the specific gut parasite *C. bombi*; since bumblebees avoid food sources contaminated by *C. bombi* more often than *E. coli* (Fig. 1). Even if they feed longer on the uncontaminated flower when contaminated by *E. coli* while this pattern is not present with *C. bombi* (Fig. 2a,3a) This result maybe an artefact from the experimental design, as short visits (<2 seconds) may have been the response time to *C. bombi* (these visits were not recorded). This is correlated with the observation of individuals tasting the nectar without landing on the flower contaminated by *C. bombi* (personal observation).

*C. bombi* is a long term and specific parasite of bumblebees resulting in co-evolution between host and parasite [20]. According to the red queen theory, it should lead to an arms race between a host and his specific parasite [43], [44], [45]. Since the bumblebee colony is composed by full-sibs, a parasite can easily spread between individuals and decrease the fitness of the entire colony [20]. Hence the adaptation of avoidance behaviour should be a decisive step with regards to parasitism in bumblebees. This hypothesis is strengthened by our results, since the presence of a non-adapted parasite toward bumblebees decrease the rewarding value of the nectar; moreover the presence of a specific parasite in the nectar leads to the avoidance of the flower. Furthermore, a bumblebee population sympatric with the *C. bombi* lineage showed a better efficiency in avoidance of contaminated flowers than an allopatric population.

Bumblebees use different cues (colours, shapes, odours of the flowers and even social cues) in order to optimise their foraging efficiency [46], [47], [48], [49], [50], [51], [52], [53], [54], [55]. These cues allow them to choose the most rewarding flowers through learning. To recognise flowers contaminated with *C. bombi* without feeding on it, bumblebees have to use cues which are perceptible before the ingestion of the contaminated sugar water. At an individual level, the most likely explanation is the presence of the odour produced directly by the parasite, which is the case in ungulates [56]. A previous study showed that bumblebees avoid flowers containing evidence of past predation events, the cues, used were the sight and the scent of a dead bumblebee [57]. A further possible cue, used to recognise the contaminated sugar water, is the taste of the sugar water Some workers were observed to extend the proboscis toward the flower and use their tongue to taste the “nectar” without landing before choosing the non-contaminated flower (personal observation).

This learning could also be the result of a colony level learning ability. The recognition of a non-contaminated flower could be provided through social cues. This could be the resultant of the use of cues from the other individuals like a copying behaviour [55] or the scent marks left on the flower [49], [53], [54]. Bumblebees leave a scent mark after visiting a flower [49], [50], [53], [54]. These scent marks can provide different information for a pollinator in regard to its previous experience [58]. Moreover, nest-mates gain cues through the odour from the successful foragers and honey pots [48]. Another social cue used by bumblebees for foraging is the copying behaviour; where bumblebees having seen a nest-mate feeding on a specific flower, will subsequently copy their flower choice [55]. Social learning is supported by our results on *C. bombi* contamination. The proportion of visits on the non-contaminated flower increased over time, while this did not occur with the contaminated flower. In addition, individuals foraging less than 5 times showed a clear preference for the non-contaminated flower without any effect from the position. Since they visit the flowers only a few times, they are not able to learn by themselves [31], [32]. This preference of naïve bees seems to result from the copying behaviour. Naïve bumblebees choose more often flowers occupied by conspecifics [59].

Our result on the individual level shows a difference between rare and frequent flyers cognitive or sensitive abilities (Fig. 4). The frequent flyers choose more often the non-contaminated flower on a first day than rare flyers did. Although, rare flyers are not so sharp on their foraging efficiency, they increased it over days showing learning. Some previous studies have demonstrated that workers from the same colony do not possess the same abilities [60], [61], [62], [63].

Another question comes into mind with regards to these results, why bumblebee population are so heavily contaminated by this specific parasite, if they are able to recognise contaminated flowers? There are many possible explanations. First the transmission of *C. bombi* can be horizontal as vertical so the parasite is also transmitted from the mother colonies to the daughters' colonies. For the horizontal transmission, the transfer of workers from a colony to another one [64], [65] could also play a preponderant role to the spread of the parasite in a population. Regarding the infection of individuals through contaminated flowers some environmental factors can mislead the bees. One could be that the odour (if the odour is the cue used by bumblebees to recognise the contaminated flower) of the flower masks or reduce the ability of bees to detect the parasite; although this is not likely due to their ability to recognise scent marks deposited by other bees on the flower [49], [50], [53], [54]. Another reason could be strong competition for food resources or a reduced availability of the optimal food source, which might force bumblebees to forage on the most rewarding flowers. The most likely explanation for this difference between our experiment and the nature is the small quantity of nectar in a natural flower (∼1 to 100 µl) compared to our flower (0.8 ml). With such small nectar quantities in the flower, the amount of *C. bombi* cells is low (compared to our experiment set-up) and should increase the difficulty for a bumblebee to detect their presence.

In conclusion, avoidance behaviour has been selected in bumblebees in order to reduce the uptake of a specific parasite when foraging on flowers. In addition they are sensitive to the presence of a common pathogen in “nectar”. The avoidance of *C. bombi* contaminated food sources appeared through learning at both, the individual and the colony level. This is mediated by the use of different cues: direct cues provided by the contamination (odour, taste, visual) and social cues provided by the other nest-mates (scent-marks, odour from honeypots and foragers, copying behaviour). These results provide a new insight on foraging strategies and resistance to parasites in bumblebees, other pollinators and social insects in general.

## Supporting Information

Figure S1 **Frequency distribution of number of flights.** The frequency of individuals in regard to their observed number of flights for the *Crithidia bombi* experiment. All replicate colonies are pooled and only the marked individuals are represented.
(EPS)

## References

[1] Bonsall MB (2004). The impact of diseases and pathogens on insect population dynamics.. Physiol Entomol.

[2] Salathé M, Kouyos RD, Regoes RR, Bonhoeffer S (2008). Rapid parasite adaptation drives selection for high recombination rates.. Evolution.

[3] Decaestecker E, Gaba S, Raeymaekers JAM, Stoks R, Van Kerckhoven L (2007). Host-parasite "Red Queen" dynamics archived in pond sediment.. Nature.

[4] Ebert D, Hamilton WD (1996). Sex against virulence: the coevolution of parasitic diseases.. Trends Ecol Evol.

[5] Cremer S, Armitage SAO, Schmid-Hempel P (2007). Social Immunity.. Curr Biol.

[6] Schmid-Hempel P (1998). Parasites in Social Insects..

[7] Shykoff JA, Schmid-Hempel P (1991). Parasites and the Advantage of Genetic Variability within Social Insect Colonies.. Proc R Soc Lond B Biol Sci.

[8] Liersch S, Schmid-Hempel P (1998). Genetic variation within social insect colonies reduces parasite load.. Proc R Soc Lond B Biol Sci.

[9] Baer B, Schmid-Hempel P (1999). Experimental variation in polyandry affects parasite loads and fitness in a bumble-bee.. Nature.

[10] Baer B, Schmid-Hempel P (2001). Unexpected consequences of polyandry for parasitism and fitness in the bumblebee, *Bombus terrestris*.. Evolution.

[11] Tarpy DR (2003). Genetic diversity within honeybee colonies prevents severe infections and promotes colony growth.. Proc R Soc Lond B Biol Sci.

[12] Tarpy D, Seeley T (2006). Lower disease infections in honeybee (*Apis mellifera*) colonies headed by polyandrous vs monandrous queens.. Naturwissenschaften.

[13] Hughes WOH, Boomsma JJ (2004). Genetic Diversity and Disease Resistance in Leaf-Cutting Ant Societies.. Evolution.

[14] Otto SP, Nuismer SL (2004). Species Interactions and the Evolution of Sex.. Science.

[15] Baer B, Schmid-Hempel P (2003). Bumblebee workers from different sire groups vary in susceptibility to parasite infection.. Ecol Lett.

[16] Biesmeijer JC, Roberts SPM, Reemer M, Ohlemüller R, Edwards M (2006). Parallel Declines in Pollinators and Insect-Pollinated Plants in Britain and the Netherlands.. Science.

[17] Cameron SA, Lozier JD, Strange JP, Koch JB, Cordes N (2011). Patterns of widespread decline in North American bumble bees.. Proc Natl Acad Sci U S A.

[18] Alford DV (1975). Bumblebees..

[19] Schmid-Hempel R, Schmid-Hempel P (2000). Female mating frequencies in *Bombus spp.* from Central Europe.. Insectes Soc.

[20] Schmid-Hempel P (2001). On the evolutionary ecology of host-parasite interactions: addressing the question with regard to bumblebees and their parasites.. Naturwissenschaften.

[21] Durrer S, Schmid-Hempel P (1994). Shared Use of Flowers Leads to Horizontal Pathogen Transmission.. Proc R Soc Lond B Biol Sci.

[22] Riddell C, Adams S, Schmid-Hempel P, Mallon EB (2009). Differential Expression of Immune Defences Is Associated with Specific Host-Parasite Interactions in Insects.. PLoS ONE.

[23] Erler S, Popp M, Lattorff HMG (2011). Dynamics of Immune System Gene Expression upon Bacterial Challenge and Wounding in a Social Insect (*Bombus terrestris*).. PLoS ONE.

[24] Alghamdi A, Dalton L, Phillis A, Rosato E, Mallon EB (2008). Immune response impairs learning in free-flying bumble-bees.. Biol Lett.

[25] Otterstatter MC, Gegear RJ, Colla SR, Thomson JD (2005). Effects of parasitic mites and protozoa on the flower constancy and foraging rate of bumble bees.. Behav Ecol Sociobiol.

[26] Jordan C, Harder L (2006). Manipulation of Bee Behavior by Inflorescence Architecture and Its Consequences for Plant Mating.. Am Nat.

[27] Popp M, Lattorff HMG (2011). A Quantitative In Vitro Cultivation Technique to Determine Cell Number and Growth Rates in Strains of *Crithidia bombi* (Trypanosomatidae), a Parasite of Bumblebees.. J Eukaryot Microbiol.

[28] R Team Core Development (2008). R: A language and environment for statistical computing.. http://www.R-project.org/.

[29] Bates D, Maechler M, Dai B (2008). lme4: Linear mixed-effects models using S4 classes.. http://cran.at.r-roject.org/web/packages/lme4/index.html.

[30] Crawley M (2005). Statistics: An Introduction using R..

[31] Riveros A, Gronenberg W (2009). Olfactory learning and memory in the bumblebee *Bombus occidentalis*.. Naturwissenschaften.

[32] Durisko Z, Shipp L, Dukas R (2011). Effects of Experience on Short- and Long-term Foraging Performance in Bumblebees.. Ethology.

[33] Schmid-Hempel P, Ebert D (2003). On the evolutionary ecology of specific immune defence.. Trends Ecol Evol.

[34] Christe P, Richner H, Oppliger A (1996). Of great tits and fleas: sleep baby sleep.. Anim Behav.

[35] Goulson D (1999). Foraging strategies of insects for gathering nectar and pollen, and implications for plant ecology and evolution.. Perspect Plant Ecol Evol Syst.

[36] Biernaskie JM, Walker SC, Gegear RJ (2009). Bumblebees Learn to Forage like Bayesians.. Am Nat.

[37] Biernaskie JM, Gegear RJ (2007). Habitat assessment ability of bumble-bees implies frequency-dependent selection on floral rewards and display size.. Proc R Soc Lond B Biol Sci.

[38] Lefebvre D, Pierre J, Outreman Y, Pierre J-S (2007). Patch departure rules in Bumblebees: evidence of a decremental motivational mechanism.. Behav Ecol Sociobiol.

[39] Bar-Shai N, Keasar T, Shmida A (2011). The use of numerical information by bees in foraging tasks.. Behav Ecol.

[40] Anfora G, Rigosi E, Frasnelli E, Ruga V, Trona F (2011). Lateralization in the Invertebrate Brain: Left-Right Asymmetry of Olfaction in Bumble Bee, *Bombus terrestris*.. PLoS ONE.

[41] Raine NE, Chittka L (2007). Flower constancy and memory dynamics in bumblebees (Hymenoptera: Apidae: Bombus).. Entomol Gen.

[42] Waser NM (1986). Flower Constancy: Definition, Cause, and Measurement.. Am Nat.

[43] Bell G (1982). The Masterpiece of Nature: the Evolution and Genetics of Sexuality; Berkeley: University of California Press.

[44] Hamilton WD, Axelrod R, Tanese R (1990). Sexual reproduction as an adaptation to resist parasites (a review).. Proc Natl Acad Sci U S A.

[45] Lively CM, Craddock C, Vrijenhoek RC (1990). Red Queen hypothesis supported by parasitism in sexual and clonal fish.. Nature.

[46] Blarer A, Keasar T, Shmida A (2002). Possible Mechanisms for the Formation of Flower Size Preferences by Foraging Bumblebees.. Ethology.

[47] Dornhaus A, Chittka L (1999). Insect behaviour: Evolutionary origins of bee dances.. Nature.

[48] Dornhaus A, Chittka L (2005). Bumble bees (Bombus terrestris) store both food and information in honeypots.. Behav Ecol.

[49] Goulson D, Chapman JW, Hughes WOH (2001). Discrimination of Unrewarding Flowers by Bees; Direct Detection of Rewards and Use of Repellent Scent Marks.. J Ins Behav.

[50] Goulson D, Stout JC, Langley J, Hughes WOH (2000). Identity and Function of Scent Marks Deposited by Foraging Bumblebees.. J Chem Ecol.

[51] Keasar T, Bilu Y, Motro U, Shmida A (1997). Foraging choices of bumblebees on equally rewarding artificial flowers of different colors.. Isr J Plant Sci.

[52] Kunze J, Gumbert A (2001). The combined effect of color and odor on flower choice behavior of bumble bees in flower mimicry systems.. Behav Ecol.

[53] Renner M, Nieh J (2008). Bumble bee olfactory information flow and contact-based foraging activation.. Insectes Soc.

[54] Saleh N, Scott A, Bryning G, Chittka L (2007). Distinguishing signals and cues: bumblebees use general footprints to generate adaptive behaviour at flowers and nest.. Arthropod-Plant Interact.

[55] Worden BD, Papaj DR (2005). Flower choice copying in bumblebees.. Biol Lett.

[56] Fankhauser R, Galeffi C, Suter W (2008). Dung avoidance as a possible mechanism in competition between wild and domestic ungulates: two experiments with chamois *Rupicapra rupicapra*.. Eur J Wildl Res.

[57] Abbott KR (2006). Bumblebees avoid flowers containing evidence of past predation events.. Can J Zool.

[58] Leadbeater E, Chittka L (2009). Bumble-bees learn the value of social cues through experience.. Biol Lett.

[59] Kawaguchi LG, Ohashi K, Toquenaga Y (2006). Do bumble bees save time when choosing novel flowers by following conspecifics?. Funct Ecol.

[60] Ings T, Schikora J, Chittka L (2005). Bumblebees, humble pollinators or assiduous invaders? A population comparison of foraging performance in *Bombus terrestris*.. Oecologia.

[61] Raine N, Chittka L (2007). Pollen foraging: learning a complex motor skill by bumblebees (*Bombus terrestris*).. Naturwissenschaften.

[62] Raine NE, Chittka L (2008). The correlation of learning speed and natural foraging success in bumble-bees.. Proc R Soc Lond B Biol Sci.

[63] Spaethe J, Weidenmüller A (2002). Size variation and foraging rate in bumblebees (*Bombus terrestris*).. Insectes Soc.

[64] Birmingham AL, Hoover SE, Winston ML, Ydenberg RC (2004). Drifting bumble bee (Hymenoptera: Apidae) workers in commercial greenhouses may be social parasites.. Can J Zool.

[65] Lopez-Vaamonde C, Koning JW, Brown RM, Jordan WC, Bourke AFG (2004). Social parasitism by male-producing reproductive workers in a eusocial insect.. Nature.

